# The speed-accuracy tradeoff: history, physiology, methodology, and behavior

**DOI:** 10.3389/fnins.2014.00150

**Published:** 2014-06-11

**Authors:** Richard P. Heitz

**Affiliations:** Department of Psychology, Center for Integrative and Cognitive Neuroscience, Vanderbilt Vision Research Center, Vanderbilt UniversityNashville, TN, USA

**Keywords:** speed-accuracy tradeoff, decision-making

## Abstract

There are few behavioral effects as ubiquitous as the speed-accuracy tradeoff (SAT). From insects to rodents to primates, the tendency for decision speed to covary with decision accuracy seems an inescapable property of choice behavior. Recently, the SAT has received renewed interest, as neuroscience approaches begin to uncover its neural underpinnings and computational models are compelled to incorporate it as a necessary benchmark. The present work provides a comprehensive overview of SAT. First, I trace its history as a tractable behavioral phenomenon and the role it has played in shaping mathematical descriptions of the decision process. Second, I present a “users guide” of SAT methodology, including a critical review of common experimental manipulations and analysis techniques and a treatment of the typical behavioral patterns that emerge when SAT is manipulated directly. Finally, I review applications of this methodology in several domains.

“… we face a very common problem in psychology: the existence of a tradeoff between dependent variables, in this case false alarms and reaction time. The only sensible long-range strategy is, in my opinion, to study the tradeoff… and to devise some summary statistic to describe it.”

- Luce, 1986, p. 56.

## Introduction

*Prima facie*, the notion of speed-accuracy tradeoff (SAT) is pedestrian. Who has not encountered that a decision, made in haste, often leads to err? Who has not felt the deleterious effects of time pressure on ultimate outcomes? The concept seems so commonsensical as to deserve little interest—an obvious product of nothing more than human limitations. Ironically, it is just this pervasiveness that demands the SAT be considered—not only as a phenomenon in and of itself—but also as a benchmark for models of the decision process. Common across task domains and in creatures ranging from house-hunting ants (Franks et al., 2003) and bumblebees (Chittka et al., 2003; for a review, see Marshall et al., 2009) to humans (Wickelgren, 1977) and monkeys (Heitz and Schall, 2012, 2013), the SAT is thus a topic of great concern. Fortunately, there has been a renewed interest in SAT, particularly in the neuroscience community. Using fMRI, EEG, and single-unit recordings, never have we been closer to understanding, at a fundamental level, how the brain takes in sensory information and transforms it into a decision variable guiding choice. As a ubiquitous phenomenon intimately tied to the decision process, the SAT is integral.

## Historical overview

The idea that response time^1^ (RT) can be used to study the inner workings of the mind is as old as psychology itself. In the mid 1800's, Hermann von Helmholtz demonstrated that peripheral nerve conduction velocity was finite and measureable—a revolutionary conception for his time. The logic was simple, yet elegant. Helmholtz created a preparation of frog legs with a portion of nerve still attached; applying current to the nerve elicited muscle contraction. He then noted the difference in the latency to contraction when either a proximal or distal portion of the nerve was stimulated. Since the distance between the stimulation points was known, Helmholtz easily worked out the conduction velocity (see Foster, 1870). Helmholtz' logic was perhaps just as important as his discovery: one can use the time of an overt movement as a dependent measure, and by altering the antecedent conditions, estimate the duration of intermediary components. Perhaps one could use similar methodology to objectively measure the component processes of the mind. This philosophy guided several researchers in their exploration of the “velocity of thought,” including Helmholtz' colleague Wilhelm Wundt, in what would be known as the first true psychology laboratory. Similar logic was employed by Merkel (1885), and very notably, by Donders (1868) in his study of processing stages using task comparisons. The use of RT—one of the only non-introspective measures available, became central.

That the accuracy of a response varies with the time taken to produce it was probably already known, if implicitly. However, such variation was of little interest, the field being dominated at either extreme by psychophysics experiments—which emphasize high accuracy without concern for RT—and reaction time experiments, which examine one's ability to produce predefined responses to simple visual or auditory stimuli. Outside of this asymptotic performance lay a nether-region of neither wholly accurate nor wholly fast responding. Still, the fact that such variability exists led some early researchers to address the speed-accuracy relation empirically. The first demonstration that the accuracy of an action varies with its speed was provided in 1899, both in a dissertation by Woodworth (1899) and a contemporaneous work by Martin and Müeller (1899), though these studies focused on the speed of obligatory movements rather than choice behavior ^2^. The first demonstration of a relationship between *choice* accuracy and decision time can be traced to 1911, when Henmon (1911) presented subjects with a simple discrimination task. Two lines were presented, each differing slightly in length, and subjects were to determine which line was longer (or shorter) and press the appropriate left or right button. In the first analysis of its kind, Henmon “binned” the data by RT to examine the effect of latency on accuracy. His data revealed an orderly relation, suggesting they were not independent. A short time later, Henmon's observations were replicated and the relationship dubbed the “speed-accuracy relation” for the first time in oft-neglected dissertation by Garrett (1922). The phenomenon received only sporadic attention thereafter, for nearly three decades.

In the intervening years, work conducted on statistical decision-making would ultimately provide a framework for understanding the SAT, and also bring the phenomenon to center stage. This work, carried out independently by Alan Turing^3^, Abraham Wald, and others, demonstrated that decision-making under uncertainty can be bolstered through sequential sampling of information—a suggestion not previously considered by the extant literature in economics (Edwards, 1954). Consider a choice between two competing hypotheses—say, whether or not a batch of product contains sufficient defects to warrant rejection. At the outset, one may already have some prior expectation regarding which hypothesis is more likely. An updated posterior probability can be computed by simply sampling information (e.g., units of product) sequentially. The problem is that information is costly—each sample takes some quanta of time and effort (Drugowitsch et al., 2012). Therefore, it is in one's best interest to sample as little as possible to reach some specified compromise between confidence and time spent sampling. Wald's procedure, which became known as the *sequential probability ratio test* (Wald, 1947), allows one to approach a known (acceptable) error rate with a potentially enormous savings in time and resources.

Turing and Wald's application was a utilitarian approach to economical decision-making, but it did not take long for others to realize that the process may apply more generally to human choice behavior. The first instance of this was provided in 1958 by Becker (1958). Participants viewed successive presentations of cards, upon each of which was an imprinted letter. Cards were drawn from one of two or more competing distributions, described to subjects prior to each run. Viewers were asked to sample as many cards as needed to determine which distribution the cards were drawn from. Becker manipulated the difficulty of the discrimination by altering the form of the parent distributions. For instance, subjects might need to determine if a sequence of “P” and “Q” letters were sampled from a distribution with a P:Q ratio of 2:1 or 1:1. Becker found that even in this abstract situation, humans produce data conforming to Wald's predictions, at least to a first approximation.

### The introduction of mathematical decision models

Meanwhile, others were working on formulating a mathematical relationship between decision time and accuracy. The first attempts, provided by Audley (Audley and Jonckheere, 1956; Audley, 1957, 1958), demonstrated that two-choice decisions could be modeled as a stochastic process. Audley had been working with albino rats trained to push one of two buttons to earn reward. At that time, stochastic models had seen success in predicting the form of the learning curve in terms of a gain in accuracy over successive trials, but they did not accommodate decision times. Nonetheless, decision times, and the RT distributions they form, were thought to reflect the structure of the choice process (Christie and Luce, 1956), and so were likely an important component of a complete choice model. Audley demonstrated that with some simple assumptions regarding the form of the underlying RT distribution (in this case, exponential), one could simultaneously predict both choice accuracy and decision time. However, the individual quanta in this situation were single, punctate choices made by rats; the model was opaque to the cognitive events carried out *within* any given trial. Audely soon remedied this, in a model that would become known as the *Runs* model (Audley, 1960); see also (LaBerge, 1962; Audley and Pike, 1965). In a guarded conceptual leap, Audley assumed that the choice process involves a series of “implicit responses” arising from the presentation of a sensory signal. Though the definition of “implicit responses” was left open to interpretation, it seems closely related to what we might now call “perceptual accumulation.” During a choice trial, observers obtain successive samples of implicit responses, and some counting mechanism keeps track of the number of consecutive runs favoring either of two potential actions. Formulated mathematically, Audley demonstrated that the model could account for choice behavior; notably, he fit the model to Henmon's data (Henmon, 1911) described earlier.

**Figure 1:**
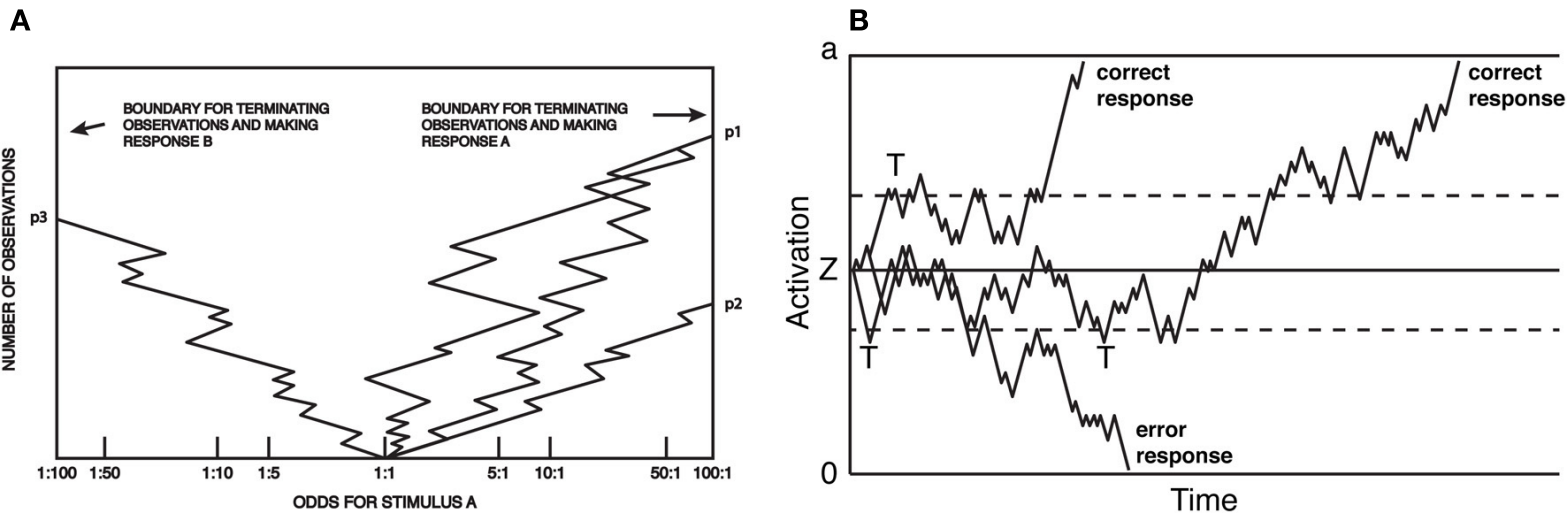
**Random-walk model of choice reaction time. (A)** Each sample can be considered evidence favoring one of two options, and at each step, the observer updates an estimate of the posterior probability (here, presented as an odds ratio) based on that evidence. A response is produced at a threshold odds ratio. Reaction time is not explicit, but proportional to the total number of samples. Adapted from Fitts (1966). **(B)** The closely related diffusion model. Here, boundaries are associated with the correct or errant response and the X-axis is real-time. As in **(A)** responses are produced when activation reaches threshold, and the SAT is a function of the placement of the threshold. Adapted from Ratcliff and Rouder (1998).

The above efforts came to a head in 1960, when Stone (1960) produced a formal mathematical model of the decision process. The model combined (1) the relation between RT and accuracy rates as a stochastic process; (2) the mathematics and optimality of the sequential probability ratio test; and (3) the presumption of information accumulation over the course of perceptual decision-making. The model, known as the *random walk*^4^, made very specific, empirically testable predictions about the means and shapes of reaction time distributions, and how those distributions change with SAT. Figure 1 presents two depictions of the random walk, adapted, respectively, from Fitts (1966) and Ratcliff and Rouder (1998). During a trial, subjects sample perceptual information, at each step computing a revised estimate of the likelihood of either hypothesis being true. Responses are produced when the observers' posterior probability exceeds some *threshold* odds ratio (Figure 1A). The same model is presented in Figure 1B, except that the process carries out more clearly in real time, and the response threshold is defined in an equivalent, yet more abstract dimension. Figure 1B illustrates how sequential sampling models implement SAT: when the decision threshold is high (solid upper and lower lines), RT tends to be longer and more likely correct, as noise in the process is allowed to average out over time. When lowered (dashed lines), the process terminates early (marked by a “T” in Figure 1B). This speeds RT, but also increases the probability that an error will result due to noise in the sampling process: note that the longest-latency correct response would result in an error under low but not high threshold. Moreover, the model makes very specific, empirically testable predictions about the form of the resulting RT distributions, and how they change with various manipulations. The random walk model received immediate acclaim, and was extended and revised almost immediately (Edwards, 1965; Laming, 1968).

The random walk model provided a rigorous and principled treatment of SAT, but was not favored by all. In Ollman (1966) proposed the first of what would become known as *mixture models*. Whereas sequential sampling models assume incremental evidence accumulation, Ollman suggested a mixture of dichotomous states: fast guesses and slow controlled decisions. The latency of the guess process and controlled process was assumed constant; SAT was achieved by simply changing the mixture. Note that this predicts a linear accuracy-RT relationship anchored by a theoretical *true guess RT* (corresponding to chance level accuracy) and a *true controlled RT* (corresponding to perfect accuracy). Intermediate values are simply weighted averages of the two component latencies. This *fast guess model* was tested by Yellott (1971). Subjects performed a simple color discrimination task while SAT was induced through *response deadlines:* arbitrary time limits subjects must beat in order to produce a fully correct response (see section SAT Manipulations). The fast guess model predicts that both unknown quantities—the true guess and true controlled RT—should be invariant over deadline conditions. Yellot devised a method for estimating these latencies, and found remarkable invariance. The guess and controlled RT was constant not only across deadline conditions, but over subjects.

The idea that SAT results from a mixture of random guesses is certainly attractive from a standpoint of simplicity. It should not be controversial that subjects can, if they wish, produce a pre-selected random guess in nearly any choice task. But, there are problems with this proposal. The most obvious is the prediction that mean error RT is faster than mean correct RT. This must occur if errors are produced by guesses, which in turn are always fast. While this is a common observation (Ollman, 1966; Schouten and Bekker, 1967; Hale, 1969; Grice and Spiker, 1979), it is certainly not the rule. Further, it is likely that Yellot's color choice task may have been so simple that subjects had to begin guessing to meet the demands of the deadline manipulation. This was in fact found to be the case. One year later, Swensson (1972a) had subjects determine which of two rectangles, oriented at 45°, was longer. SAT was induced using a payoff matrix that favored accurate or fast responding. Swensson conducted a regimented trial-by-trial analysis, categorizing each as a likely guess or non-guess response. When the discrimination was simple, Swensson found data consistent with the fast guess model: subjects either used a guessing strategy or a highly accurate controlled strategy. A mixture also obtained when the discrimination was made more difficult, except for one critical detail. When the analysis was limited to non-guess trials, accuracy rate continued to vary with RT. Swensson proposed an alternative, known as the *deadline model* ^5^. Like the fast guess, subjects are assumed to mix pure guesses with correct responses, but whether or not a guess is to be made is not decided prior to the trial. Instead, subjects maintain an internal timer; SAT manipulations set a limit on this timer. A response is produced either when sufficient information has been gleaned as to make a correct response, or when the deadline is reached. While intuitively appealing, the deadline model has seen little success. For one thing, one might better term the model the *slow guess*, as it predicts error RTs that are later than correct RTs—a prediction not borne out by Swensson's own data and numerous other studies (but see Estes and Wessel, 1966; Pike, 1968; Link and Tindall, 1971; Audley, 1973; Pfefferbaum et al., 1983; Ditterich, 2006a; Heitz et al., 2010). Perhaps more problematic for the deadline model—indeed all mixture models—is the observation that error RT is sometimes faster and sometimes slower than correct RT (Link and Tindall, 1971; Swensson, 1972a; Luce, 1986). Mixtures models are not flexible enough to predict both. Other efforts have rendered mixture models untenable as a sole explanation for SAT (Reed, 1973; Ruthruff, 1996; Wagenmakers et al., 2008; but see Dutilh et al., 2011; Schneider and Anderson, 2012; Donkin et al., 2013).

For several reasons, sequential sampling has emerged as the dominant decision model framework. For one, they naturally account for choice behavior under SAT without appeal to a mixture of two states, and with some assumptions, can predict either fast or slow error RT (Laming, 1968; Ratcliff and Rouder, 1998). Another is precision: they provide a quantitative account of mean correct and error RT, accuracy rate, the shapes of correct and error RT distributions, and how each of these change with experimental manipulations such as SAT, response bias, and the strength of sensory evidence. Third, they make testable predictions. For instance, when sensory evidence remains constant, there exists a unique, optimal decision threshold that maximizes reward rate (RR) (Gold and Shadlen, 2002; Bogacz et al., 2006), and humans closely match this threshold even when optimality changes between blocks of trials (Simen et al., 2009; Bogacz et al., 2010a; Balci et al., 2011). Likewise, these models can be shown to account for high-level behaviors such as visual fixations and purchasing decisions (Krajbich et al., 2010, 2012; Milosavljevic et al., 2010; Towal et al., 2013). Fourth, there is mounting evidence that something akin to sequential sampling occurs in the brain, as I will discuss later.

There exist several sequential-sampling models that embrace these strengths, notably, the Drift-Diffusion (Ratcliff, 1978; Busemeyer and Townsend, 1993; Ratcliff and Smith, 2004), Race/Accumulator (Pike, 1968; Vickers and Smith, 1985; Smith and Vickers, 1988; Logan, 2002), Leaky-Competing Accumulator (Usher and McClelland, 2001), LATER (Carpenter and Williams, 1995; Reddi and Carpenter, 2000), and Linear Ballistic Accumulator (Brown and Heathcote, 2005, 2008) among others (cf. Cisek et al., 2009; Drugowitsch and Pouget, 2012; Thura et al., 2012; Thura and Cisek, 2014). Though a full discussion is beyond the scope of this article [the reader may refer to Bogacz et al. (2006) and Ratcliff and Smith (2004)], it should be noted that nearly all assume SAT is a function of the distance (or “excursion,” Churchland et al., 2008) a decision variable must travel from a start point to a threshold, sometimes called *response caution* (Forstmann et al., 2008). In many, SAT is implemented by a change in decision threshold alone (Figure 1). This idea has been challenged, and several efforts now consider SAT to be a multifaceted phenomenon including changes in, for example, sensory gain (Ditterich, 2006b; Standage et al., 2011, 2013; Heitz and Schall, 2013) along with decision threshold.

### Summary

The SAT has long been a phenomenon of interest in behavioral science. From early on, the covariation between response speed and accuracy was seen not as a nuisance, but a signature of the decision process itself. Consequently, experimental investigations of SAT progressed largely in parallel with mathematical models of the decision process. This work is ongoing, but a consensus has emerged: agents make choices based on a sequential analysis of sensory evidence. As decades of research make clear, this decision process is adaptable: actions are dictated not only by the nature of perceptual input but also environmental constraints, internal goals, and biases. An embodiment of this flexibility, the SAT arises due to the inherent contradiction between response speed and decision accuracy. Faster responses entail less accumulated evidence, and hence less informed decisions. Sequential sampling models provide an intuitive framework for understanding SAT. Observers set a decision criterion—an amount of evidence required to commit to a choice—based on current task demands and internal goals. This begs the question: how can we know *what* decision criteria subjects employ? It would seem that without this knowledge, mean RTs and accuracy rates conflate experimental factors with strategic effects employed by the observer. The solution to this problem is to bring decision criterion^6^ under experimenter control. As explained below, this not only avoids ambiguity, but also quantifies *precisely* how accuracy trades off with latency.

## SAT methodology: experimental manipulations and analysis techniques

A common theme in the above is the manipulation of subjects' decision criteria through experimenter influence. These *SAT experiments* quantify how accuracy covaries with RT over the range of decision criteria subjects might use. In contrast, group means obtained at a single criterion provide only a snapshot of performance that conflates decision strategy with the nature of the task (e.g., its difficulty). In other words, with decision criteria free to vary, many different group means could obtain, from very fast RT and chance accuracy to very slow RT and asymptotic accuracy. The problem is further exacerbated if the experimental conditions under comparison also encourage different SAT settings, making group means difficult to interpret and conclusions ambiguous (Wickelgren, 1977; Lohman, 1989). In this way, SAT manipulations avoid problems shared by *non-SAT experiments*, echoed in the quote that opened this work. Furthermore, deriving the pattern of performance over a variety of decision criteria, SAT experiments offer a window into the decision process itself. An empirical example will drive home the point.

Heitz and Engle (2007) addressed the possibility that individuals rated high or low on a measure of working memory capacity exhibit differences in processing efficiency during low-level visual (non-memory) tasks. Specifically, they proposed that those with low working memory process sensory evidence more slowly than those with high working memory capacity. To test this, high and low working memory subjects performed the Eriksen flanker task (Eriksen and Eriksen, 1974; Gratton et al., 1988). Subjects reported the identity of a central letter (H or S, mapped to key presses on different hands), each flanked on either side with response-congruent or response-incongruent stimuli. Subjects typically respond more quickly and with higher accuracy to congruent (e.g., HHHHH) than incongruent (e.g., HHSHH) strings. Heitz and Engle manipulated SAT through the use of response deadlines ranging from 200 to 700 ms. By implicating rate of perceptual accumulation, they predicted that asymptotic performance would be equivalent. That is, if given sufficient time, both groups should perform equally. This is particularly suited for SAT methodology, as obtaining group means at a single criterion would not address the question.

**Figure 2:**
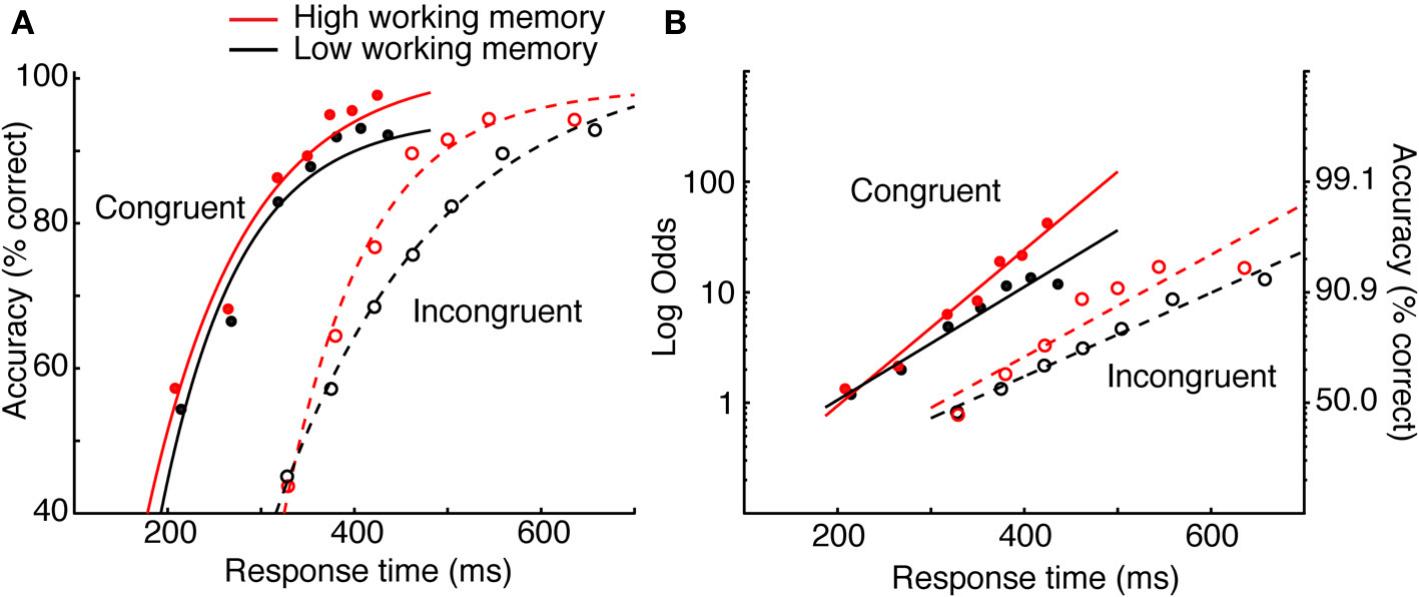
**Data from Heitz and Engle (2007) Experiments 1 and 2. (A)** Data were fit by an exponential approach to a limit. The critical pattern concerns the incompatible condition (dashed lines). The groups do not differ in intercept or in asymptote, but do differ in rate. **(B)** The same data in **(A)** linearized using a log-odds transformation and fit with a log-linear regression.

The data in Figure 2A depict accuracy rate conditionalized on RT ^7^ (known as a *conditional accuracy function*—a topic I will return to). The data are fit by a function known as an *exponential approach to a limit*^8^, as is common (Wickelgren, 1977; McElree and Dosher, 1989; Öztekin and McElree, 2010), to obtain numerical estimates of intercept (the processing time needed to make above-chance, informed decisions), rate (gain in accuracy with RT), and asymptote (peak accuracy). The critical pattern concerns the difference between high and low working memory groups on incompatible trials (dashed lines). It is observed that at very fast RT, both groups are equally fast and at respond at about chance level. Asymptotic accuracy also appears equivalent, suggesting that the two groups perform equally when given sufficient time. What distinguishes the groups is the rate of gain in accuracy with RT, which the authors interpreted as evidence that the groups did in fact differ in processing efficiency. The relationship is perhaps more straightforward when the negatively accelerated function is linearized using a log-odds transformation, also a common practice (Figure 2B). It is clear that the slope of the function relating accuracy and RT is greater for the high than low working memory group. This conclusion—quite different than the authors had expected—was made possible though SAT manipulations^9^. In sum, bringing decision criteria under experimenter control provides a detailed picture of the decision process, avoids ambiguity that may arise when SAT is not controlled, and facilitates more specific hypotheses. Numerous experimental methods accomplish this, each with strengths and weaknesses.

### SAT manipulations^10^

#### Verbal instructions

In the vast majority of behavioral studies, subjects are directed to maintain both high accuracy and fast RTs. This is problematic, as the two constraints are contradictory. As pointed out humorously by Edwards (1961): “These instructions are internally inconsistent. A computing machine would reject as insoluble a problem presented with such instructions” (p. 276). It is with this in mind that Howell and Kreidler carried out the first true SAT experiment (Howell and Kreidler, 1963). In a task similar to the venerable Hick paradigm (Hick, 1952), different groups of participants were asked to favor fast, accurate, or fast and accurate responding. In their own words, this required “… that *S* establish a “trade-off” between two dimensions” (p. 41). For obvious reasons, instructions remain the most common SAT manipulation: they are simple to implement, require little training, and yield large effect sizes.

Though popular, verbal instructions are not ideal in several respects. First, instructions are qualitative. It is unlikely that individuals adopt similar response criteria both within and between emphasis conditions (Lohman, 1989), which serves to both diminish effect sizes and increase experimental error (Edwards, 1961). Moreover, without a quantitative method, the potential for regression to the mean is high. Subjects may modify behavior initially, but over the course of trials in a block, settle into some less distinct mode. In fact, there is a tendency for controlled RT distributions to skew toward an individual's natural mean RT (Schouten and Bekker, 1967). Second, the number of qualitatively different emphasis conditions subjects can achieve is limited; any more than three seems difficult. This is certainly adequate for gross comparisons (e.g., Hale, 1969; Osman et al., 2000; Forstmann et al., 2008; Ivanoff et al., 2008), but may be inadequate for describing the accuracy-latency function mathematically, particularly if decision criteria are not homogenous over subjects (McClelland, 1979). Finally, and particularly important for future work, instructions are decidedly not available in non-human subject populations.

***Payoffs***. To combat the ambiguity of instructions, Fitts (1966) designed a payoff matrix to differentially reward correct decisions and penalize errors. Fitts defined four response categories, based on whether the response was correct and whether the RT met an arbitrary “criterion time.” As shown in Table 1, subjects were awarded +1.0 point for fast and correct responses, and penalized −1.0 point for slow and inaccurate responses. The SAT emphasis conditions were distinguished by the penalty incurred for correct but slow or incorrect but fast responding. Under accuracy emphasis, there was a higher penalty associated with errors, whereas under speed emphasis, the penalty was greater for slow responding. This scheme worked quite well; payoff matrices induced significant covariation in RT and accuracy rate even in the absence of verbal instructions. Others have since used similar methods to manipulate SAT (Pachella and Pew, 1968; Swanson and Briggs, 1969; Lyons and Briggs, 1971; Swensson and Edwards, 1971; Gehring et al., 1993).

**Table 1:** **Payoff matrices used by Fitts (1966) to induce SAT**.

**Payoff condition**	**Correct and fast**	**Correct and slow**	**Wrong and fast**	**Wrong and slow**
Pretest	+1	−0.2	−0.2	−1
Speed	+1	−0.5	−0.1	−1
Accuracy	+1	−0.1	−0.5	−1

Payoffs have at least two advantages over verbal instructions. First, the quantitative nature of the rewards and penalties allow for a larger number of emphasis conditions. Secondly, verbal instructions become unnecessary; observers learn contingencies over the course of the experiment or in practice blocks, making this method viable for use with non-human populations. On the other hand, the payoff scheme requires one to define a “criterion time” that defines whether or not a particular response is considered fast or slow. Ideally, the criterion time is determined subject-by-subject using a data-driven method, such as some percentile of a subjects' RT distribution during the same task without time constraints. Whether arbitrary or subject-specific, the choice of the criterion time separating “fast” and “slow” RT is an important consideration, as improper values render the method ineffective. That said, some early studies have seen success using a constant, arbitrary criterion time for all subjects (Fitts, 1966; Ollman, 1966; Pachella and Pew, 1968). It is also worth noting that without additional instructions or cuing events, switching between emphasis conditions will not be immediate.

***Pure payoffs***. Avoiding the problem of arbitrary criterion times, Swensson designed a method making rewards and penalties linearly related to RT (Swensson and Edwards, 1971). Correct responses are rewarded [D − *k*(RT)] and errors penalized [−*k*(RT)]. Parameter *k* specifies the relative gain or loss with changes in RT, while D defines the relative gain due to correct responding. When D is small, rewards and penalties are based entirely on RT; when large, the reward associated with correct responding outweighs loss due to long latency. This regime, known as “pure payoffs,” has seen little use (Swensson and Edwards, 1971; Swensson, 1972a,b), but is in principle superior to a standard payoff structure. Unfortunately, it shares one weakness with the payoff matrix: learning the reward contingencies takes time, and subjects will be unable to switch between conditions immediately without ancillary cuing signals.

***Deadlines***. Pachella introduced a simplification of the payoff procedure described above. He demonstrated that SAT can be induced using only the criterion times that define “fast” and “slow” responses without any associated payoff matrix (Pachella and Pew, 1968; Pachella and Fisher, 1969). As is typical, a single deadline is in effect throughout a block of trials; choice latencies that do not beat the deadline are met with some tone or visual feedback to indicate the response was not fast enough^11^. Practice trials preceding each block provide an acclimation period. Numerous classic and contemporary works use this simple, highly effective manipulation (Pachella and Pew, 1968; Pachella and Fisher, 1969; Link and Tindall, 1971; Yellott, 1971; Green and Luce, 1973; Pike et al., 1974; Jennings et al., 1976; Ratcliff and Rouder, 2000; Diederich and Busemeyer, 2006; Heitz and Engle, 2007; Yamaguchi et al., 2013).

There are several considerations that warrant discussion. The first is the number of deadline conditions, which depends on both the desired resolution as well as willingness to obtain increasingly more observations per subject. While as few as three are sufficient to mathematically describe the tradeoff function (McClelland, 1979), as many as 5–8 are not uncommon (Schouten and Bekker, 1967; Yellott, 1971; Jennings et al., 1976; Heitz and Engle, 2007). In regards to selecting particular deadline values, it is important to have an idea of both the mean and variance of subjects' RT during an unconstrained version of the same task. One then selects *N* deadlines that more than span this range. Note that spanning too large a range increases experimental complexity with diminishing returns. Deadlines that are too fast will encourage guessing, and deadlines that are too long will have little to no effect. Another concern is the order of the deadline blocks. If all subjects are presented with the same order, practice effects become confounded with SAT effects. It is desirable to present deadlines in random or pseudo-random order, ideally with multiple repetitions to account for gains in performance over time.

***Deadline tracking***. An even more principled method for manipulating SAT uses an adaptive tracking method coupled with a deadline procedure. Rinkenauer et al. (2004) targeted particular accuracy rates (97.5, 82.0, 66.0%) instead of RT *per se.* Accuracy rate was computed in successive blocks of trials, and deadline values increased or decreased (in 30 ms steps) accordingly. This data-driven method has the advantage of naturally accounting for practice effects, attentiveness, fatigue, etc. that may alter behavior throughout an experiment. However, because accuracy rates must be computed over sets of trials, there is considerable overhead in converging to a desired performance level. Furthermore, if practice effects are large, substantial changes in the underlying RT distributions may occur despite holding average accuracy rate constant.

***Response-to-stimulus interval (RSI)***. In the absence of explicit SAT manipulations, subjects are thought to choose decision criteria that maximize potential reward, whether that be monetary or otherwise (Edwards, 1965; Gold and Shadlen, 2002). One's RR is simply the proportion of correct responses divided by the average length of a trial. Several factors contribute to the average length of a trial (and hence RR), including decision time, non-decision related (e.g., sensory) delays, and the interval between one's response and the beginning of the following trial (the *response-to-stimulus interval*, RSI). Recent theoretical work suggests that altering RSI should provide a means to implicitly alter one's SAT criteria (Bogacz et al., 2006). This makes intuitive sense: when RSI is long and the pace of the task is slow, the available number of decision opportunities is likely to be fewer than when RSI is short and the pace is fast. In this case, the optimal RR is attained through slow, highly accurate decision-making. Conversely, when RSI is short, the optimal RR is achieved by emitting decisions more quickly, even if many of those decisions are incorrect. This has firm empirical support: RSI manipulations lead to SAT in much the same way as conventional time limitations (Simen et al., 2009), and mathematical decision models localize the effect to decision threshold (Simen et al., 2006; Bogacz et al., 2010a; Balci et al., 2011)^11^.

The use of RSI to manipulate SAT has several advantages. First, it is divorced from any explicit time limitations and is clearly a voluntary, strategic adaptation. Second, RSI is formalized mathematically in decision models and makes contact with a theoretical literature on RR optimization. Third, RSI may be ideal for use with non-human populations. On the other hand, RSI manipulations do not take effect immediately, as observers cannot optimize decision criteria instantaneously (Simen et al., 2009; Balci et al., 2011). Even the most sensitive subjects may require as many as 20 trials before performance stabilizes, and not all subjects produce an effect (Bogacz et al., 2010a). Furthermore, the assumption that RSI operates on subjects' inherent motivation to maximize RR seems to require experimental designs that are time-limited rather than trial-limited. In practice, this point may be moot as subjects appear to remain sensitive to RSI even in fixed trial length blocks (Simen, personal communication, 4/3/2014).

***Response signals***. The last two methods, *response signals* and *RT Titration* are motivated by different goals. Whereas the methods above attempt to alter subjects' cognitive state, the following attempt to bring RT under experimental control while keeping SAT criteria constant. The response signal method^12^ was first developed in 1973, as a direct test of the *fast guess* model (Reed, 1973). The procedure effectively prevents fast guesses by allowing subjects to respond only when cued; in this case, the disappearance of visual stimuli served as the signal. Even with fast guesses eliminated, Reed observed that accuracy rate covaried with RT, rendering the fast guess model untenable.

The strength of this method lies in the unpredictable nature of the upcoming trial. The duration of the stimulus-to-cue duration cannot be anticipated, ensuring that each trial is approached with equivalent cognitive states—exactly the opposite intention as instructions, deadlines, etc. In this case, the accuracy-latency relationship is less likely to involve strategic changes in decision criteria but rather results from the quantity of information accumulated before encountering the cue to respond. Early cues truncate processing and force a response based on partial information.

There are two weaknesses to this approach. First, for long cue delays, subjects may withhold their response when they would otherwise have emitted a choice. In sequential sampling terms, responses are obligated not by threshold crossing but by external influence, questioning its relevance to the normal choice process. (Even the deadline method allows the choice process to terminate normally on most trials.) Related to this point, the choice process has been altered such that one cannot be sure exactly *what* SAT criterion observers are using. The method simply ensures that, on average, observers use the same criterion at the beginning of each trial, or alternatively, that the criterion does not vary in any controlled way. The last method obviates this concern.

***RT titration***. RT Titration (Meyer et al., 1988) seeks to hold constant observers' SAT criteria trial-to-trial while ensuring subjects begin each trial as if it were a normal, no-signal, free RT task. The procedure is straightforward: subjects make choices whenever they wish, unless a response signal is encountered, at which time a response is obligated. Because many trials include no response signal, behavior on each trial is governed by the same sequential sampling process in operation during non-SAT tasks. Meanwhile, the influence of processing time on accuracy and the contribution of partial information can be gauged by those trials including a response signal. In many ways, RT Titration is superior to the response signal method, except that subjects require training in order to produce responses swiftly after encountering the relatively more rare response signal.

Methods that hold decision criteria constant (response signals and RT Titration) are fundamentally different from those that force criteria to change (instructions, deadlines, etc.). Must the form of the accuracy-latency relationship also be different? One study to test this compared the deadline and response signal methods in the same subjects during the same task (Dambacher and Hübner, 2013). Interestingly, there was surprising agreement between the two, despite a tendency for lower overall performance in the response signal method. How can there be so much agreement between such disparate methodologies? This can be explained by the constancy of the perceptual input. Whether perceptual accumulation terminates naturally due to threshold crossing or is truncated artificially by experimenter influence, the stimulus information driving the process remained constant. What *does* differ—and this may partially explain the discrepancy between the methods—is that the predictable deadline procedure allows for proactive adjustments, such as the type observed in the baseline neural firing rates in monkeys (Heitz and Schall, 2012). Additionally, the response signal method likely involves extra cognitive demand as observers must also perform signal detection.

***Selecting the best SAT manipulation***. All of the above methodologies are effective, but which is most appropriate? The answer is guided by at least three considerations: (1) should RT be explicitly controlled; (2) should decision criteria vary between conditions; and if so (3) must adjustments be immediate? A guide is presented in Table 2, but is non-exhaustive. For instance, verbal instructions might be combined with deadlines to ensure at least minimal control of mean RT (e.g., Forstmann et al., 2008), making it an instance of “explicit” RT control. Likewise, the response signal method will allow decision criteria to vary if presented in a blocked format (Schouten and Bekker, 1967).

**Table 2:** **Summary of SAT methodologies**.

**RT control**	**Decision criteria**	**Adjustment time**	**Method**
Indirect	Altered	Fast	Verbal instructions
Indirect	Altered	Slow	RSI
Explicit	Altered	Fast	Deadlines
Explicit	Altered	Slow	Payoffs, Pure payoffs, Deadline tracking
Explicit	Invariant	–	Response signals, RT Titration

### Analysis of speed-accuracy tradeoff data

There are several methods for depicting the SAT; here I deal with the three most popular: the *speed-accuracy tradeoff function* (SATF), the *conditional accuracy function* (CAF), and the *quantile-probability plot* (QPP). To facilitate the discussion, I created a simulated SAT experiment employing three response deadlines at 225, 325, and 425 ms. The manipulation was assumed effective, with mean accuracy rates increasing linearly at 50, 70, and 90%, respectively. RT distributions for each condition were generated by drawing 10,000 observations randomly from an ex-Gaussian (van Zandt, 2000) distribution (σ = 20 ms, τ = 30 ms) such that approximately 25% of all RTs fell later than the RT deadline in each condition, but these “missed deadlines” were not removed. The mean RT for error trials was set to be slightly (5 ms) faster than correct trials.

#### SATF

**Figure 3:**
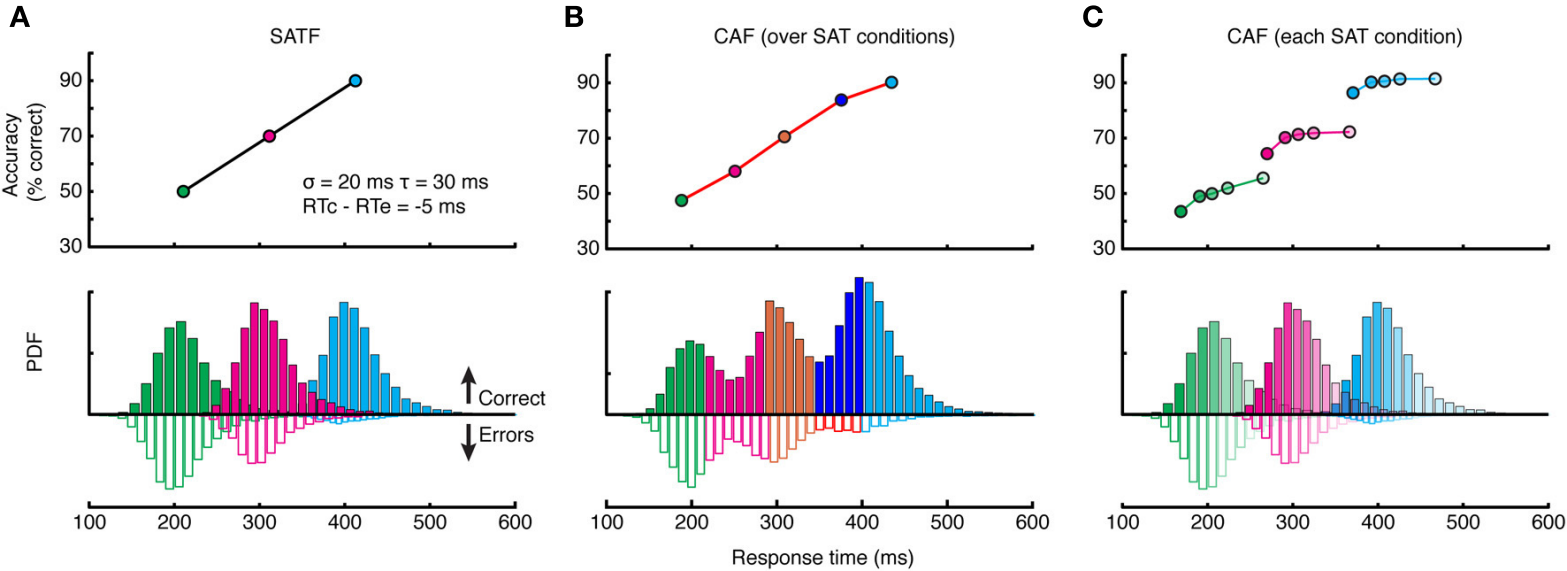
**Comparison of the SATF, overall CAF, and individual CAFs in the same simulated dataset. (A)** The SATF is simply the mean RT and accuracy rate for each SAT condition Here, they were 225, 325, and 425 ms response deadlines. The manipulation was effective by definition, yielding accuracy rates of 50, 70, and 90%, respectively. Each condition was constructed by drawing *N* = 10,000 observations randomly from an ex-Gaussian distribution with parameters indicated in figure inset. Solid histograms depict correct trials, open histograms error trials. **(B)** The same data as **(A)** but aggregated disregarding SAT condition and plotted as a CAF. **(C)** Same data as **(A,B)** but CAFs computed separately for each deadline condition.

The SATF plots mean RT and accuracy rate for each SAT condition separately (Figure 3A). It reflects the efficacy of the experimental manipulation and quantifies how accuracy trades off with RT, on average. The SATF is robust to the variability of the component distributions: the extent to which conditions overlap has no effect, nor is it influenced by the direction of mean error RT. However, it is clear that there is considerable variation within each condition not captured by the SATF. For instance, observed RTs of ~250 ms obtain in both the shortest and middle deadlines. Are these responses qualitatively different? Restated, the question is whether or not the large-scale difference between SAT conditions (the *macro-SAT*) is due to the same factor as smaller-scale, within-condition variability (the *micro-SAT*; Pachella, 1974; Thomas, 1974; Wood and Jennings, 1976; Wickelgren, 1977; Grice and Spiker, 1979; Vickers et al., 1985). Perhaps the difference in between- and within-condition variability is only one of magnitude; the macro-SAT due to large changes in decision criteria and micro-SAT due to intrinsic variability and trial-to-trial adjustments (Ridderinkhof, 2002; Jentzsch and Leuthold, 2006).

#### CAF

If this were the case, it makes more sense to plot accuracy rate conditionalized on observed RT disregarding deadline condition altogether. All RT data are categorized into equal-observation quantiles, and accuracy rate is computed separately for each bin (Figure 3B). Though this provides a more detailed description of how accuracy trades off with RT, this *overall* CAF does not address whether similar latencies collected under different deadline conditions are psychologically equivalent. This may be accomplished by computing CAFs *individually* for each deadline condition (Figure 3C). If the micro- and macro-SAT have the same source, the SATF, CAF, and individual CAFs should be overlapping (but see Grice and Spiker, 1979).

**Figure 4:**
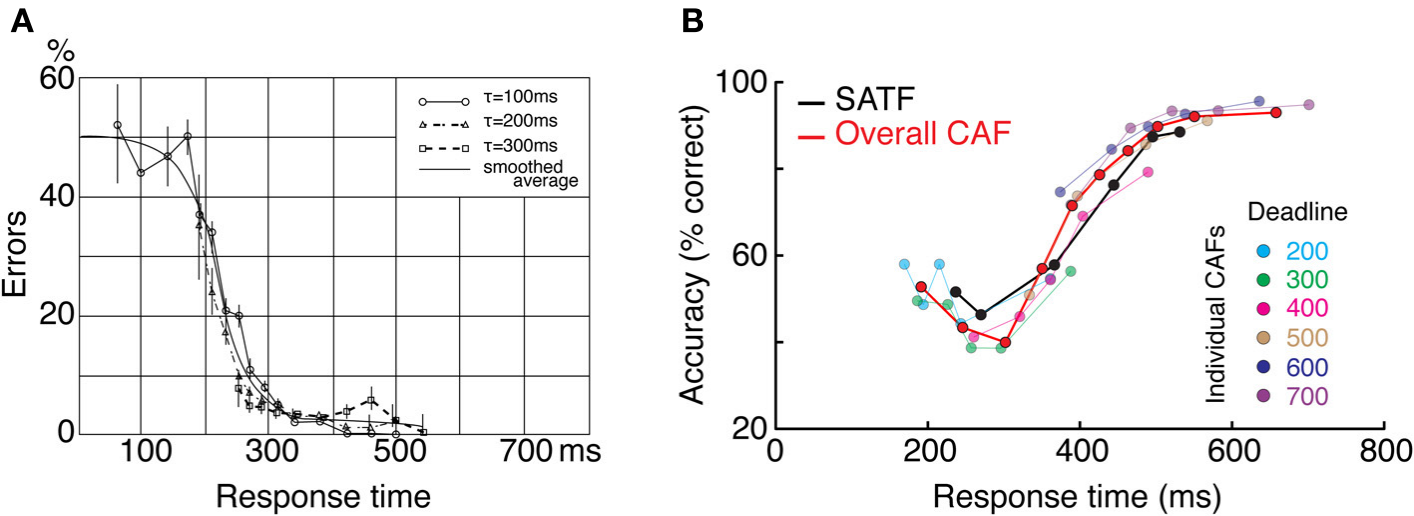
**Two empirical examples when the CAF—both the overall CAF and individual CAFs overlap with the SATF. (A)** (Schouten and Bekker, 1967) forced subjects to respond to respond at target RTs during a simple two-choice response time experiment. They found that the individual CAFs overlapped significantly; the accuracy rate associated with a given RT was invariant with respect to the forced response time condition. The overall CAF and SATF are approximated by the black ogive running through individual points. Data were traced using graphics software from the original work. Note that error rate (rather than accuracy rate) is plotted on the y-axis. **(B)** (Heitz and Engle, 2007) presented subjects with a two choice response compatibility experiment under 6 response deadlines. These data, replotted from their incompatible condition, clearly indicate gross agreement between the SATF (black), overall CAF (red), and individual CAFs (colored lines). Based on this agreement, these authors used the overall CAF as their primary measure to retain time resolution.

**Figure 5:**
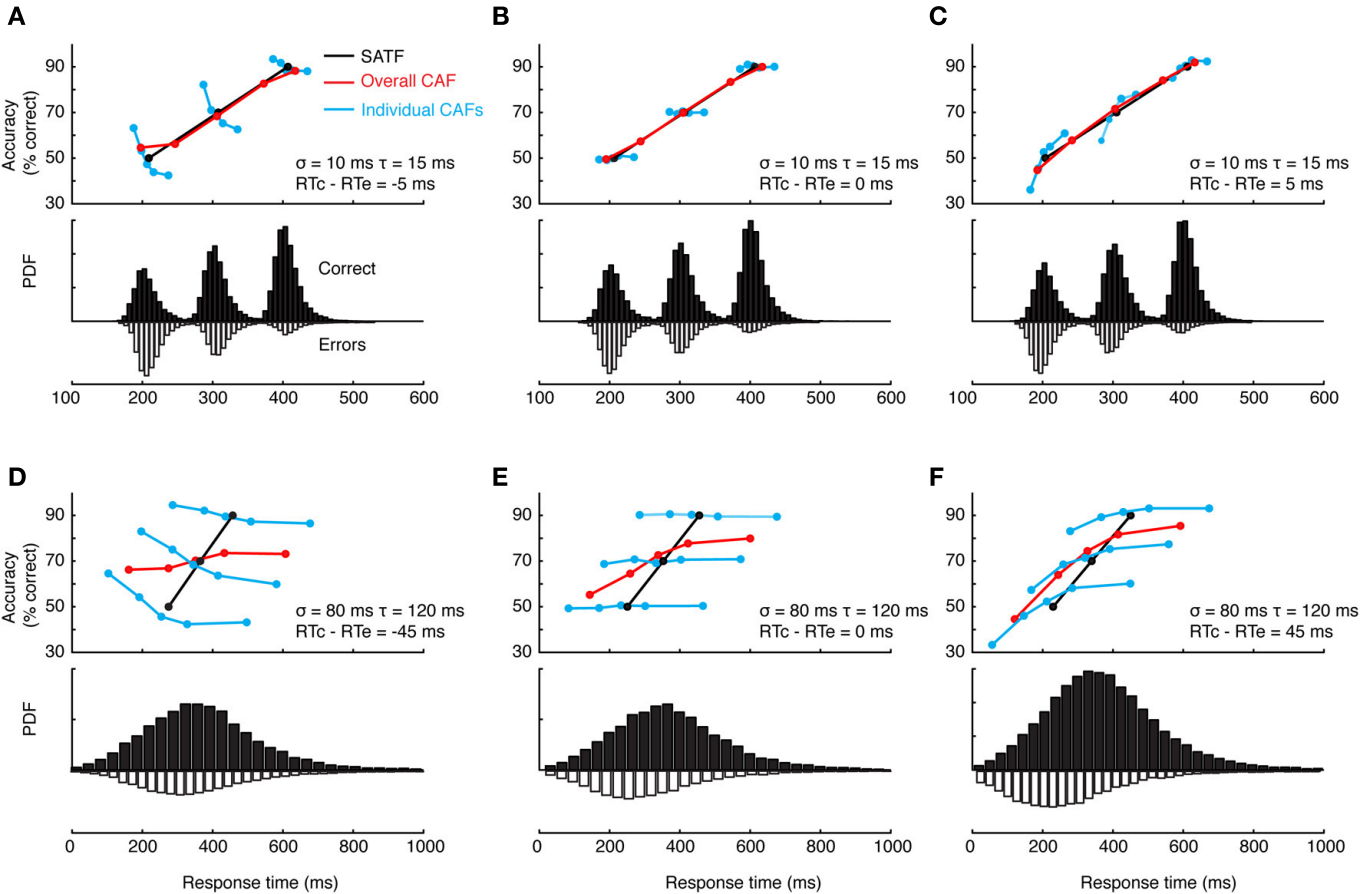
**Dependence of the CAF on component RT variance and direction of mean vs. correct RT. (A–C)** With small RT variability, distributions exhibit little overlap, leading the overall CAF (red lines) to be a fair representation of the SATF with better resolution in time. The form of the individual CAFs (blue) are dictated by the direction of correct and error RT, exhibiting a downward trend for slow errors **(A)** a flat line with equal mean correct and error RT **(B)** and an upward trend for fast errors **(C)**. **(D–F)** The mismatch between SATF, overall CAF, and individual CAFs is exaggerated with more reasonable parameters. When RT distributions significantly overlap, the overall CAF no longer reflects the SATF.

This can and does occur—two examples are presented in Figure 4—but it is perhaps more common to find that they disagree. The reason for this becomes apparent when two parameters are varied—the extent of overlap between the RT distributions and the direction of mean error RT. To demonstrate, I repeated the simulation described above while manipulating both the variability (and tail) of the RT distributions and the direction of mean error RT, being faster, equal, or slower than mean correct RT (Wood and Jennings, 1976). The results are presented in Figure 5. In the top row (Figures 5A–C), the standard deviation of the distributions is kept small, so as to include little overlap between the SAT conditions. In this unrealistic situation, the overall CAF is a fair representation of the SATF, but the individual CAFs may be decreasing **(A)**, flat **(B)**, or increasing **(C)** depending on the direction of mean error RT. It is straightforward to understand why: when error RTs are slower than correct RTs, early quantile bins necessarily contain more correct than error responses **(A)**. If mean RTs are equal **(B)**, each bin will on average contain the same number of errant and error-free trials. Finally, when mean error RT is faster than correct **(C)**, early bins will tend to be less accurate, and later bins more accurate. The pattern is exaggerated in the more realistic situation of extensive overlap between RT distributions (Figures 5D–F). In this case, neither the overall CAF nor individual CAFs approximate the SATF. It would seem that the CAFs are unpredictable and dominated by the simple direction of mean error RT. This is true, but beside the point. While all sequential sampling models predict an increasing SATF, the form of the micro-SAT differs. For instance, the original random walk model (Stone, 1960) predicts flat CAFs, since correct and error RT are equivalent (Pachella, 1974). In contrast, some accumulator models (Vickers et al., 1985) and the random walk with collapsing bounds (where threshold decreases over time) predict decreasing or inverted “U” shape CAF (Pike, 1968). Increasing CAFs are consistent with several models, including the fast guess (Pachella, 1974), variable criterion model (Grice et al., 1977), some versions of the random walk (Laming, 1968; Vickers et al., 1985), and others.

#### Quantile probability plots

**Figure 6:**
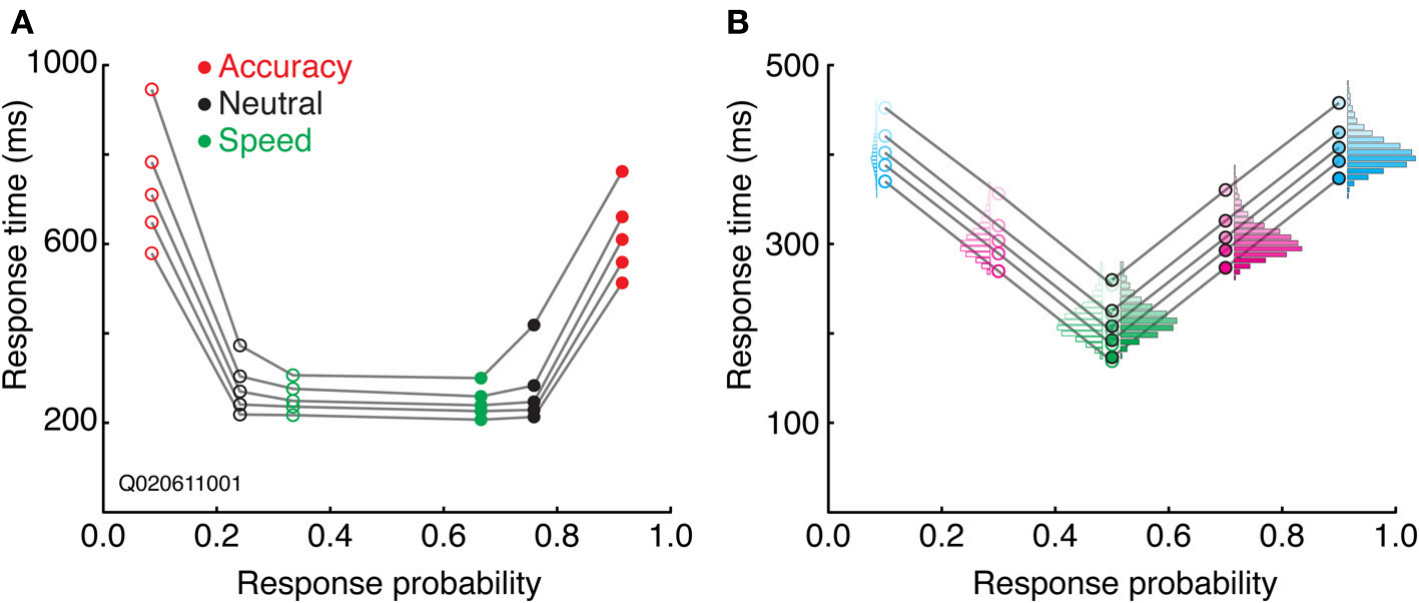
**Quantile-probability plots. (A)** The QPP calculated from a single non-human primate during an SAT task. Open points to the left of 0.5 correspond to errors, closed points to the right of 0.5 are correct trials. Each vertically oriented set of 5 points mark the RT quantiles described in the text. Lines connect quantiles between SAT conditions (red = Accuracy stress, black = Neutral, and green = Speed stress). **(B)** The QPP calculated from the same simulated dataset presented in Figure 3. The individual-condition RT distributions (Figure 3C) are reflected in the quantiles of the QPP.

Combining aspects of both the SATF and CAF is the *quantile probability plot* (Audley and Pike, 1965; see also Ratcliff and Tuerlinckx, 2002). The SATF and CAF describe changes in accuracy rate with RT, but do not depict distributional characteristics, aspects that are particularly important in evaluating the fit of mathematical decision models (Audley and Pike, 1965; Pike, 1968). The drift-diffusion model, for instance, makes quantitative predictions regarding the shape of correct and error RT distributions; the QPP describes this information succinctly. For each condition, RT quantiles are calculated separately for correct and error trials, commonly at the 10, 30, 50, 70, and 90th percentiles. The RT corresponding to these quantiles are then plotted against response probability for each condition. For instance, if the accuracy rate for a particular condition was 80%, the RT quantiles for correct trials would be plotted at 0.8, and corresponding error trials at 1.0 − 0.8 = 0.2. Under most circumstances, points to the left of 0.5 represent error trials and those to the right of 0.5, correct trials (but see Simen et al., 2009). A typical QPP computed on SAT data from a single (non-human primate) subject (Heitz and Schall, 2012) is shown in Figure 6A. Several characteristics are apparent. First, both accuracy rate and RT tend to increase from a Speed emphasis condition to an Accuracy emphasis condition, giving the QPP a “U” shape. This convexity is diagnostic: in sequential sampling models such as the drift-diffusion, increasing decision bounds lead to a slowing of RT with an increase in accuracy rate. In contrast, a concave QPP indicates that accuracy rate is improving while RT becomes faster, a common occurrence when signal quality is manipulated (Ratcliff and Smith, 2010). Second, error RT tends to be longer than correct RT. The difference is small in the Speed and Neutral conditions (note the one point in the Neutral condition not following this trend), but quite large in the Accuracy condition. Third, the spread of the RT distributions increase with SAT stress, as might be expected given the large changes in mean RT. Fourth, the distribution of error RTs appears roughly equivalent to correct RTs in the Speed and Neutral conditions, but noticeably larger for error trials in the Accuracy condition, particularly in the tail. The QPP provides a wealth of information absent in the SATF and CAF, yet they are related. Figure 6B displays this relation using the same simulated data as that of Figure 3.

#### Selecting the best analysis technique

There is no one best depiction of SAT, as each of the methods described above present different information, but there are guidelines. The SATF is the most common and straightforward approach, assuming only that the experimental design included some type of SAT manipulation. The QPP provides further detail, but requires a more sizeable dataset: estimation of RT quantiles becomes unreliable when trial counts are low, and this can be particularly problematic when errors are rare. The QPP has the additional benefit of being closely related to mathematical decision models, but less clearly depicts the rate of gain in accuracy with RT.

Overall CAFs, computed across an entire dataset, are only appropriate in specific situations. First, in the context of non-SAT experiments, the CAF might be computed to evaluate subjects' natural tendency to trade speed for accuracy (Lappin and Disch, 1972a,b) and is indeed the only available option. Second, when the CAF and SATF are overlapping, the former leads to the same conclusion as the latter while providing slightly more resolution on the RT axis (Figure 4). Individual-condition CAFs are useful in assessing the direction of error RT on a fine scale, but are rarely used as a sole dependent measure.

#### Summary

The use of SAT methodology continues to offer insight into the decision process, and how that process is altered strategically. The above provide numerous routes for obtaining and depicting the SAT. Unfortunately, SAT experiments are costly relative to non-SAT experiments, most requiring several times the number of observations. Is this gain in precision really worth the investment? In what follows, I briefly review domains outside of cognitive psychology where this has proven true.

## Applications of SAT methodology

### Neural activity under SAT

A fundamental question in cognitive neuroscience concerns how the brain adapts to bring about strategic changes in decision criteria. The SAT is pervasive, and behavioral changes often large; certainly brain activity must manifest a signature of SAT. The answer to this question offers insight into the neural basis of an elementary cognitive operation, and also bears on the viability of mathematical decision models.

The sequential sampling framework described earlier has recently graduated from an abstract cognitive model to an assumed neural reality—a viable method the brain may use to carry out perceptual decision-making. Evidence supporting this claim derives from several sources, including human fMRI (Heekeren et al., 2004) and EEG (Ratcliff et al., 2009; O'Connell et al., 2012; van Vugt et al., 2012; Kelly and O'Connell, 2013), but by far the most convincing stems from single-neuron recordings in non-human primates. In the typical paradigm, monkeys view a display of static or dynamic stimuli that requires a perceptual discrimination and subsequent choice between alternative actions. Their decision is communicated through an eye movement or button press, and juice reward is delivered when the response is correct. Strikingly, activity in frontal eye field (Hanes and Schall, 1996; Kim and Shadlen, 1999; Woodman et al., 2008; Ding and Gold, 2012), lateral intraparietal area (Roitman and Shadlen, 2002; Gold and Shadlen, 2007), and superior colliculus (Horwitz and Newsome, 1999; Ratcliff et al., 2007) exhibits patterns closely resembling the sequential sampling process. Most germane is the fact that neural activity grows over time during the deliberation period and terminates at a fixed threshold at the moment an overt decision is produced. In accordance with the model, much of the variability in RT can be accounted for by the duration of the firing rate excursion—the time taken to ramp from a baseline to a fixed threshold. Further lending credence, computational (Ditterich, 2006a; Purcell et al., 2010, 2012; Zandbelt et al., 2014) and neural network models (Lo and Wang, 2006; Wong et al., 2007; Beck et al., 2008; Wang, 2008; Zhang and Bogacz, 2010; Drugowitsch et al., 2012) inspired by the sequential sampling process capture both behavior and neurophysiology while respecting biological constraints. The neural activity associated with SAT is thus a topic of great concern, and has been examined using several techniques.

#### fMRI

A number of studies have used fMRI to examine neural activity during SAT manipulations. Though an fMRI approach to SAT suffers in several respects (Stark and Squire, 2001; Logothetis, 2008; Bogacz et al., 2010b), it is notable that all agree on at least one conclusion: SAT manipulations affect more than decision threshold. In fact, the most consistent finding is that relative to accuracy emphasis, placing subjects under speed stress leads to an increase in the BOLD response during *baseline* intervals (Forstmann et al., 2008; Ivanoff et al., 2008; van Veen et al., 2008; Bogacz et al., 2010b). This would seem to be interpretable within the sequential sampling framework by positing that baseline shifts are functionally identical to threshold shifts—either ultimately affects the amount of information accumulated prior to decision^13^. More interesting is the observation that more than one factor changes under SAT; at least one fMRI study has implicated changes in sensory processing with SAT (Ho et al., 2012). Further complicating the story, SAT manipulations appear to affect BOLD in region-specific ways (Vallesi et al., 2012), sometimes in opposing directions (Blumen et al., 2011). This calls into question the generality of the process: does sensory integration occur simultaneously and interactively amongst brain regions, or is there independence among sites of integration (Zhang, 2012)?

#### EEG

Unlike fMRI, EEG does not suffer from temporal blurring, but does not offer opportunity to definitively localize brain regions. Despite this, EEG components accurately track attention and error monitoring (Woodman and Luck, 1999; Heitz et al., 2010; Godlove et al., 2011), the chronometry of action preparation (Gratton et al., 1988), and the temporal evolution of the decision process (O'Connell et al., 2012; Kelly and O'Connell, 2013; van Vugt et al., 2014). In one early study, Gehring et al. (1993) examined the error-related negativity (ERN) under SAT using a deadline procedure. The ERN is a fronto-central negativity that appears in the moments surrounding error commission (Nieuwenhuis et al., 2001) and is though to reflect the error monitoring process. When accuracy was emphasized, the magnitude of the ERN was greater than under speed stress, when errors mattered less. This finding suggests that in addition to altering the decision process, SAT affects post-decision processing as well.

Several other studies sought to identify the processing stage locus of SAT: does speed stress affect early sensory processing or later decision and motor processing? Unfortunately, this issue remains unresolved. The first attempt to address this—in fact the first study to record neural activity under SAT—used the P3 component during a line length discrimination task under speed or accuracy emphasis (Pfefferbaum et al., 1983). The latency of the P3, thought to mark the completion of stimulus processing, was earlier under speed than accuracy stress, suggesting that early perceptual processing was indeed facilitated. The next attempts used the *lateralized readiness potential* (LRP), a component that tracks the evolution of motor preparation. Two studies using the LRP have concluded that SAT manipulations do not affect sensory processing (Osman et al., 2000; van der Lubbe et al., 2001; see also Wenzlaff et al., 2011), while a third demonstrated that it affects both early and late processing stages (Rinkenauer et al., 2004).

Each of the above studies examined the average EEG component time-locked to some event of interest, but there is much more information in the raw signal than is immediately apparent. Understanding this, at least one study has examined the effect of SAT on the EEG frequency spectra (Pastötter et al., 2012). Using a two-choice discrimination task, subjects were cued trial-by-trial to emphasize speed or accuracy. They found that, during the baseline interval in which SAT emphasis was cued, the EEG tended to oscillate more in the lower frequency bands (4–25 Hz) under accuracy emphasis than speed emphasis (see also van Vugt et al., 2012; Heitz and Schall, 2013).

#### Single-unit neurophysiology

To date, there has been only one single-unit recording study employing SAT manipulations (Heitz and Schall, 2012). Monkeys were trained to perform saccade visual search under Accuracy, Neutral, or Speed emphasis, cued by the color of a fixation point. Meanwhile, neural activity was recorded from the frontal eye field, a key region in the planning and execution of eye movements. The results were diverse but can be described succinctly: SAT cues affected several stages of information processing, and speed stress generally amplifies neural activity rather than attenuate it. This was most evident for baseline neural activity (increasing under Speed stress during the pre-trial interval) and in the sensitivity of neurons to visual stimulation (responding more vigorously under Speed stress). This indicates that SAT emphasis affects perceptual processing, a suggestion that has recently gained support (Standage et al., 2011; Ho et al., 2012; Thura et al., 2012; Dambacher and Hübner, 2014; Rae et al., 2014). Surprisingly, neural threshold—the level of activity reached at saccade decision—was greater for speed than accuracy emphasis, opposite the assumption of sequential sampling models. In further analyses, it was shown that SAT affects much more than the firing rates of neurons, including the extent to which single neurons were coupled with their surrounding neural network (spike-field coherence), as well as the sensitivity of that network (Heitz and Schall, 2013).

#### Summary

The coupling of SAT methodology and neuroscience techniques has the potential to offer real insight into the neural mechanisms supporting decision. The consensus emerging suggests that SAT is a multifaceted phenomenon, influencing several components of the decision process and accompanied by distinct changes in brain activity. It is interesting to suppose that external changes in brain function—due to drugs, pathology, and senescence—might lead to distinct declines in cognitive performance. SAT methodology will be particularly useful in pinpointing the locus of the deficit. The next section reviews this modest, but promising literature.

### SAT with drugs and pathology

Cognitive impairments often accompany drug use, disease, injury, and pathology. For instance, individuals with schizophrenia and certain types of brain injuries exhibit impulsive, perseverative behavior on measures such as the Wisconsin Card Sort and antisaccade tasks (Guitton et al., 1985; Fukushima et al., 1988; Kane and Engle, 2002; Thakkar et al., 2011; Cutsuridis et al., 2014). Likewise, monkeys permitted to self administer cocaine over long periods of time demonstrate increased impulsivity and reduced ability to switch between task sets (Liu et al., 2008, 2009). In contrast, aging is associated with lower performance and longer latencies (Salthouse, 2012), some of which is thought to be a “general slowing” of cognition (Kail, 1991). Do these populations simply differ in decision criteria, or has the information processing system been affected, and if so, how? A handful of studies have employed SAT methodology to address these questions.

#### Drugs

There have been few studies of SAT under the influence of controlled substances. The most extensively tested is the effect of alcohol. SAT was manipulated using instructions (Tiplady et al., 2001) or response deadlines (Jennings et al., 1976; Rundell and Williams, 1979) while subjects were given graded doses of alcohol and asked to perform auditory or visual discrimination tasks. In each case, alcohol reduced the slope of the SATF in a dose-dependent manner. As was the case of high and low working memory capacity described earlier (Figure 2), this suggests a reduction in the rate of information processing. In a more recent study, subjects performed dot motion discrimination under placebo, moderate dose, or high dose of alcohol. No SAT manipulation was included. Application of the drift-diffusion model localized the effects of alcohol to two components: drift rate and non-decision time, suggesting that perceptual accumulation was both degraded and delayed with increased intoxication (van Ravenzwaaij et al., 2012).

In other work, monkeys administered graded doses of the NMDA antagonist ketamine demonstrated both slower and more accurate performance during visual search, indicating that decision criteria may have been altered (Shen et al., 2010). Finally, a few studies have assessed the effects of stimulants on information processing, but results are inconclusive. In one, low doses of nicotine administered to non-smokers was found to benefit information processing in the absence of any SAT (Le Houezec et al., 1994). In another, the dopamine agonist bromocriptine was found to have no effect (Winkel et al., 2012) while other work suggests the dopamine reuptake inhibitor methylphenidate alters decision criteria but does not benefit information processing (Carlson et al., 1991).

#### Pathology and age

Research dealing with patient populations suggests a deficit in the information processing system itself rather than non-optimal decision criteria. In schizophrenics for instance, at least one modeling study suggests that relative to controls, patients suffer from increased sensory noise (Cutsuridis et al., 2014) and one explicit SAT study provides anecdotal support (Schweitzer and Lee, 1992). Similar conclusions are reached for Parkinson's Disease patients (Wylie et al., 2009). Interestingly, the situation is quite different for one patient group of particular interest: attention-deficit hyperactivity-disorder (ADHD). Relative to controls, ADHD subjects exhibit SATFs that are shifted, but not different in slope (Sergeant and Scholten, 1985a,b) suggesting that the rate of information processing is equivalent. Recent work suggests that ADHD patients instead have a relative inflexibility in optimizing decision criteria (Mulder et al., 2010; but see Metin et al., 2013).

There is a well-characterized decline in cognitive functioning with age, but exactly what component of the decision process is altered remains unclear. On one hand, older adults tend to be more considered and cautious in their responding (Forstmann et al., 2011), suggesting a tendency to use higher decision criteria than their younger counterparts. Indeed, modeling studies suggest that older adults fail to set decision criteria optimally, often preferring overall accurate performance at the cost of speed (Phillips and Rabbit, 1995; Ratcliff et al., 2004; Starns and Ratcliff, 2010, 2012). Empirical studies using SAT methodology corroborate this, but also provide compelling evidence for an impairment in information processing (Salthouse, 1979; Madden and Allen, 1991; Hertzog et al., 1993; Kumar et al., 2008) see also (Myerson et al., 2007).

#### Summary

Though the cognitive impairments accompanying drug use, pathology, and age are well characterized, the underlying basis remains elusive. Traditional experimental approaches cannot dissociate performance changes due to strategic effects (e.g., preference for fast than accurate decisions) from those due to information processing *per se* (e.g., compromised perceptual sampling). By placing SAT criteria under experimental control, the true nature of the deficit becomes clear. Further research will be enlightening, and may be the key to developing targeted interventions.

## SAT in non-human organisms

The present work has primarily dealt with human behavior; in stark contrast, this final section reviews a handful of studies assessing SAT in non-human populations (monkeys, rodents, and insects). This short discussion has two motivations. First, I wish to promote the use of SAT methodology in populations amenable to single-unit recordings. Neuroscience approaches continue to elucidate the decision process with unparalleled detail, and single-unit recordings are arguably the most definitive. This effort has been limited by the absence of methods for controlling decision criteria in non-human populations; here I show it is possible. Second, I wish to illustrate that the SAT is truly universal. Unlike humans and monkeys (and probably rodents), social insects also exhibit SAT, but in a very different way. Specifically, the decision to be made is one involving a colony, rather than a single member. Likewise, whereas many individual neurons contribute to a single decision in higher species, many individual entities contribute to a group decision in insect colonies. Whether or not these phenomena are comparable remains to be seen, but important parallels exist.

### Monkeys

There has been only one study using experimenter-induced SAT in monkeys (Heitz and Schall, 2012). Monkeys performed saccade visual search and were induced to respond at three levels of SAT emphasis: speed, neutral, and accuracy. Conditions were signaled by the color of a fixation point and presented in blocks of 10–20 trials. Emphasis conditions were defined by differential reward and punishment (time-out) contingencies, and monkeys were trained until they adapted behavior immediately upon presentation of a new emphasis condition. In several ways, the SAT in monkeys is identical to that in humans: the SATF is increasing, and the behavior is well fit by sequential sampling models with changes in decision threshold between emphasis conditions. There are slight differences, however. Whereas humans most commonly exhibit fast errors during visual search, monkeys tend to commit slow errors, leading to a decreasing (rather than increasing) CAF. Interestingly, this occurs even in tasks that do not include SAT manipulations, such as standard form visual search (Heitz et al., 2010) and the venerable random dot motion paradigm (Roitman and Shadlen, 2002; Ditterich, 2006a; Churchland et al., 2008). The origin of this disparity is not understood, but has not been systematically studied.

### Rodents

Evidence for SAT in rodent models is mixed. Using olfactory discrimination, one study has shown a lack of any relationship between accuracy rate and decision time, even when odor mixtures are highly similar (Uchida and Mainen, 2003; see also Zariwala et al., 2013; but see Abraham et al., 2004). However, a different conclusion emerges when the stimulus-sampling period is placed under experimenter control. One such study used an analog of the response signal method. During an olfactory discrimination task, mice were required to continue sniffing until an auditory buzzer signaled the availability of reward (Rinberg et al., 2006). The resulting SATFs were undeniably similar to that of humans. Moreover, the slope of the accuracy-latency relationship was altered by task difficulty: when odors were highly similar, the rate of gain of accuracy with RT was much lower than for highly dissimilar, and therefore easier, discriminations (see also Brunton et al., 2013).

### Insects

There is some evidence for SAT in bumblebees trained to perform a type of visual search task: bees are rewarded with sucrose for choosing to land on a target “flower” presented amongst distractors. Commonly, the flowers are distinguishable through color, but other times through scent. Like humans, bees produce linear speed-accuracy relationships (Chittka et al., 2003; Kulahci et al., 2008; Riveros and Gronenberg, 2012). Those that decide more slowly tend to be more accurate than those that respond quickly. Also like humans, changing task parameters can lead to shifts of the accuracy-latency function. For instance, when errant choices are met with punishment (quinine solution), individual bees slow down and increase accuracy relative to conditions without penalty (Chittka et al., 2003). Other manipulations that lead to SAT in bees include difficulty of discrimination (Dyer and Chittka, 2004; Skorupski et al., 2006; Riveros and Gronenberg, 2012) and the introduction of environmental stressors such as predation risk (Ings and Chittka, 2008).

Like many social insect colonies, bees choose nesting cites based on quorum sensing (Seeley and Visscher, 2004). Briefly, scout bees examine potential locations for hives and recruit others; the colony as a whole “decides” to migrate to the nest when a quorum threshold (QT) has been reached (Passino et al., 2008). It is interesting to note the parallel between the QT and the decision threshold described by sequential sampling models. Under a lower QT, fewer bees contribute to the choice of nesting cite increasing the potential for err. A computational model of bee quorum sensing confirms that changing the QT (the number of bees needed to commit to the new hive) implements SAT in an ecologically valid way (Passino and Seeley, 2006).

I am not aware of any empirical study testing this assertion in bees, but it is certainly true for ants. Like bees, ants that have found a potential nesting cite recruit others until a QT is reached. At threshold, the colony switches from individual exploration into a mode of “social carrying” in which ants pick up and carry other ants to the new cite. The SAT becomes evident when the QT is examined under different conditions. For instance, ant colonies lower their QT when placed in a harsh environment necessitating migration (Franks et al., 2003, 2009), relative to a calm environment. Similarly, QT is lowered when nests are destroyed, leading to emergency migration (Dornhaus et al., 2004); see also (Marshall et al., 2006). Interestingly, this reduction in QT has the consequences expected with SAT—faster, but less discerning migration decisions.

### Summary

The SAT is a truly universal phenomenon. Monkeys and rodents can be trained to vary decision criteria on cue, and exhibit behavior similar to humans. Future studies employing SAT methodology with these populations will provide critical insight into the decision process. There are parallels, too, with social insect colonies, and this has not gone unnoticed. These ecologically valid studies speak to the mechanisms of emergent behavior through the interaction of individual entities.

## Conclusion

The SAT has been a topic of great concern for over a century. Throughout its history and still today, the SAT remains an integral component of empirical, theoretical, and mathematical explorations of the decision process. The growing popularity of SAT in the neuroscience community is particularly exciting. The last decade has witnessed incredible advances in our understanding of the neural basis of choice, and neural investigations of SAT are now gaining momentum. This work promises to detail the choice process—not just in humans but non-humans as well—and will find utility in understanding and treating common cognitive deficits. Clearly, there is much work to be done. To facilitate this, and to bring together disparate literatures and disciplines, the present work reviewed the history, methodology, physiology, and behavior associated with SAT.

### Conflict of interest statement

The author declares that the research was conducted in the absence of any commercial or financial relationships that could be construed as a potential conflict of interest.

## Footnotes

^1^The term “response time” and “reaction time” are typically used interchangeably, and I will make no distinction here, but there is a slight semantic difference. “Reaction time” is often associated with the limits of ability, as in making a fast, predetermined response to the onset of a visual stimulus, whereas “response time” more generally describes “time to overt action.” See (Luce, 1986).

^2^As the present work is focused on choice behavior, the movement speed-accuracy tradeoff will not be considered. The reader is referred to (Hancock and Newell, 1985; Meyer et al., 1990; Plamondon and Alimi, 1997).

^3^Turing's effort was directed at breaking the Nazi enigma machine. For a fascinating review, see (Gold and Shadlen, 2002).

^4^The random walk process is by no means limited to psychology, but has seen application in physics, chemistry, and economics. It was first proposed by Pearson (1905), the same year that Albert Einsten published work on the closely related, continuous-time stochastic process describing Brownian motion, later to become known as the diffusion process.

^5^The deadline model is usually attributed to Swensson (1972a), but it was in fact proposed earlier, both by Nickerson (1969) as well as an alternative to his own fast guess model by Yellott (1971).

^6^I use the terms SAT setting, SAT criterion, and decision criterion equivalently to refer to one's momentary willingness to trade response speed for accuracy. It is a single point along an accuracy-latency performance function (Wickelgren, 1977; Lohman, 1989). In the context of sequential sampling models, it is often referred to as decision threshold.

^7^Data are collapsed over Experiments 1 and 2 of (Heitz and Engle, 2007). For fitting, initial RT-accuracy bins with chance-level responding were eliminated. The conclusions remain unaltered. See original publication for details.

^8^The exponential approach to a limit takes the form: *Acc* = λ [1−*e^−γ(T−δ)^*] where *Acc* is some measure of accuracy rate (proportion correct or d-prime), λ is asymptotic performance, γ the rate, δ the x-axis intercept, and *T* is RT. The use of an exponential approach to a limit has been criticized (Ratcliff, 2006) on the grounds that it is atheoretical and not necessitated by process models such as the drift-diffusion. Others might consider this a strength.

^9^For a similar application of SAT methodology to memory phenomena, see (McElree and Dosher, 1989; Kumar et al., 2008; Öztekin and McElree, 2010).

^10^Several of the below SAT methodologies were previously reviewed by Wickelgren (1977).

^11^How to treat “missed deadline” trials is an important issue. On one hand, it can be argued that missed deadline trials are qualitatively different from made deadline trials (e.g., subjects failed to adopt the appropriate decision criterion), and so might be eliminated. On the other hand, this leads to artificially truncated RT distributions and artifactual effects on mean RT and accuracy rate. The most conservative approach is to compute mean RT and accuracy rate for each condition as if deadlines did not exist (i.e., categorically accurate responses count as correct even when deadlines were not met). In practice, overall conclusions are robust to this choice.

^12^Interestingly, human subjects seem to perform sub-optimally, with accuracy rates slightly too high and and mean latencies slightly too long to maximize RR (Simen et al., 2009; Bogacz et al., 2010a). Why this is so is not fully understood, but it is worth noting that humans can learn to become optimal with sufficient practice (Balci et al., 2011).

^13^There is actually an earlier example. In 1967, Schouten and Bekker presented subjects with a simple choice task and cued them to respond on the last of three acoustic “pips” (but not earlier). Critically, the duration of the stimulus-to-cue interval was blocked, such that subjects would adopt different SAT settings. In this sense it is similar to the deadline manipulation, except that early responses are not allowed.
